# Application of acute myocardial infarction-related key genes in noninvasive diagnosis: a comprehensive analysis based on transcriptome and single-cell transcriptome

**DOI:** 10.3389/fgene.2026.1828693

**Published:** 2026-07-02

**Authors:** Mingbin Xie, Yuanhong Wu, Xinyao Jin, Fengchun Jiang, Qiang Yao

**Affiliations:** Department of Cardiology, Hangzhou Red Cross Hospital, Hangzhou, Zhejiang, China

**Keywords:** acute myocardial infarction, biomarker, diagnostic model, single-cell RNA sequencing, WGCNA

## Abstract

**Background:**

Acute myocardial infarction (AMI), a highly fatal cardiovascular emergency, presents ongoing clinical challenges in both early diagnosis and the elucidation of its associated immuno-inflammatory processes. By integrating multi-omics data, this research seeks to identify novel diagnostic biomarkers and uncover their potential mechanisms in AMI.

**Methods:**

By integrating transcriptomic data, core genes linked to AMI were identified by differential expression and weighted gene co-expression network analysis (WGCNA). A multivariate logistic regression diagnostic model was constructed based on these genes, and its diagnostic efficacy was evaluated with receiver operating characteristic (ROC) curve analysis. *In vitro*, an oxygen-glucose deprivation/reperfusion (OGD/R) model was generated in AC16 human cardiomyocytes. We used lentiviral transduction to knock down the key gene. Cell proliferation was evaluated by Cell Counting Kit-8 (CCK-8) assay, apoptosis levels were measured by Terminal deoxynucleotidyl transferase dUTP nick end labeling (TUNEL) staining and flow cytometry, and the mRNA and protein expression of key genes were detected with qRT-PCR and Western blot.

**Results:**

Three AMI core genes (PLA2G15, ADAP2, and FAM20C) were successfully identified. The diagnostic model established via multifactorial logistic regression exhibited satisfactory discriminatory power. *In vitro* experiments confirmed that ADAP2 expression was markedly upregulated in the OGD/R model. Knocking down ADAP2 effectively alleviated OGD/R-induced suppression of cell proliferation, apoptosis enhancement, and expression increase of inflammatory factors (TNF-α, IL-6, IL-1β). Mechanistically, ADAP2 knockdown inhibited the expression of key proteins in the IL-17 signaling pathway (IL-17A, IL-17RA, ACT1). Exogenous addition of rhIL-17A reversed the protective effect of ADAP2 knockdown against cellular injury.

**Conclusion:**

The combined diagnostic model derived from the AMI core genes PLA2G15, ADAP2, and FAM20C exhibited robust diagnostic power for noninvasive diagnosis. ADAP2 can influence AMI-induced myocardial injury through the IL-17 signaling pathway.

## 1 Introduction

Acute myocardial infarction (AMI) is a predominant cause of disability and death worldwide. It primarily results from the rupture of a coronary atherosclerotic plaque and subsequent acute thrombus formation, resulting in an abrupt cessation of blood flow (Mlynarska et al., 2024; Joseph and Yusuf, 2023). The resulting severe ischemia and hypoxia in the myocardial tissue rapidly trigger metabolic disturbances in cardiomyocytes, culminating in their programmed necrosis and irreversible injury (Du et al., 2025). The current clinical management of AMI centers on achieving timely reperfusion, supported by pharmacological therapies including antiplatelet agents, anticoagulants, and lipid-lowering drugs for plaque stabilization (Saito et al., 2023). However, existing therapeutic strategies remain limited in halting the progression of myocardial injury and enhancing repair capacity (Saito et al., 2023). The search for novel biomarkers in AMI is imperative to enable early non-invasive diagnosis, optimize risk stratification, and ultimately improve clinical outcomes.

The current diagnostic approach for AMI centers on serial electrocardiogram (ECG) changes and elevated cardiac enzymes. Although these biomarkers exhibit high specificity, they still present certain limitations in early diagnosis, identification of micro-infarctions, and differentiation from non-ischemic chest pain (Muzyk et al., 2020; Machanahalli Balakrishna et al., 2023). Consequently, the investigation of biomarkers for earlier and precise detection of myocardial ischemia, as well as the development of corresponding diagnostic models, holds considerable clinical implications for optimizing AMI patient management. For instance, one study developed a T-cell exhaustion signature-based diagnostic model, which utilized five key genes to achieve early and precise identification of AMI and revealed underlying immune microenvironment disturbances (Jin et al., 2024). The support vector machine (SVM) model developed from the identified risk genes exhibited a strong ability to discriminate between AMI patients and healthy controls, yielding a marked improvement in diagnostic performance (Song et al., 2021). However, current models predominantly rely on single-omics data, leaving a gap in the comprehensive integration of multidimensional information spanning bulk transcriptomics and single-cell resolution. Additionally, the general applicability and stability of these models in clinical practice have yet to be sufficiently verified. Therefore, constructing a diagnostic model capable of integrating key molecular signatures and reflecting the immune characteristics of AMI is important for enabling timely and accurate disease identification and revealing its pathological mechanisms.

The immune system orchestrates a continuum of responses during cardiac remodeling, contributing to both tissue repair and maladaptive pathology. The extensive loss of cardiomyocytes induced by AMI exceeds the heart’s compensatory and repair capacity. Simultaneously, damage-associated molecular patterns released by necrotic cells activate a potent innate immune response, eliciting a substantial inflammatory cascade (Algoet et al., 2023). This response subsequently prompts necrotic cardiac cells to release many stress signals, including pro-inflammatory cytokines and chemokines. These signals recruit and activate various immune cells, guiding the sequential infiltration of neutrophils, monocytes/macrophages, and subsequently T cells, B cells, and natural killer cells into the infarcted area (Kologrivova et al., 2021). Myocardial infarction induces trained immunity in monocytes, which, via epigenetic reprogramming, maintains a pro-atherogenic phenotype and accelerates the progression of atherosclerosis (Dong et al., 2024). Furthermore, a single-cell sequencing study revealed that monocyte-derived macrophages in the heart after AMI differentiate into two major subsets: pro-inflammatory and lipid-associated Trem2hi populations. Their dynamic transition and spatiotemporal distribution jointly regulate the balance between repair and inflammation following cardiac injury (Rizzo et al., 2023).

Through multi-omics integrative analysis, this study elucidates the key molecular events underlying the pathogenesis of AMI. We seek to develop a clinically applicable, non-invasive diagnostic model and to investigate the mechanisms by which key genes modulate immune responses and cell differentiation. This work lays the groundwork for advancing early diagnostic methods and immunomodulatory treatments for AMI.

## 2 Methods

### 2.1 Data acquisition

Microarray and scRNA-seq data were obtained and screened from the Gene Expression Omnibus database (https://www.ncbi.nlm.nih.gov/geo/). The GSE269269 dataset, containing peripheral blood samples from 5 patients with STEMI, was utilized for scRNA-seq analysis. For RNA-seq analysis, this study employed the GSE62646, GSE141512, and GSE59867 datasets. The GSE62646 dataset included peripheral blood samples from 14 patients with stable coronary artery disease (CAD) and 84 patients with ST-elevation myocardial infarction (STEMI). The GSE141512 dataset comprised samples from 6 CAD patients and 6 STEMI patients. The GSE59867 dataset contained peripheral blood samples from 46 CAD patients and 111 STEMI patients.

### 2.2 Differential gene expression analysis

Differential expression analysis of the AMI transcriptome data from the GSE62646 and GSE141512 datasets was conducted with the “limma” R package (Wang et al., 2023). The screening threshold was set at |logFC| > 0.25 and an adjusted p-value <0.05. For the scRNA-seq data, differential gene analysis was conducted using the FindMarkers function from the “Seurat” package (Tan et al., 2023). The Wilcoxon rank-sum test was employed to compare differences between groups, with a threshold set at logfc. threshold >0.25 and *p* < 0.05.

### 2.3 Weighted gene Co-expression network analysis (WGCNA) of microarray data

Based on the GSE62646 dataset, WGCNA was performed with the “WGCNA” package (Wang et al., 2023) in R. For network construction, the 5,000 most highly expressed genes in the AMI cohort were extracted. During the construction of the weighted adjacency matrix, a soft thresholding power of 10 was applied to ensure the network satisfied a scale-free topology model (fit index R^2^ ≥ 0.83). Based on this criterion, the threshold yielding a scale-free topology fit closest to or above 0.8 was thereby chosen to generate a stable, interpretable network. Subsequently, hierarchical clustering analysis was performed to calculate the topological overlap measure (TOM) among genes and to assign genes into different modules. Module identification employed a “hybrid” dynamic tree-cutting method, with parameters set as follows: a merge cut height of 0.4 and a minimum module size of 60 genes. Finally, analysis of the TOM further revealed gene-gene interactions and potential functional associations within the network.

### 2.4 Identification of core genes for AMI

To identify core AMI genes, the MEpink module genes derived from WGCNA were overlapped with the differentially expressed genes (DEGs) from the GSE62646 and GSE141512 cohorts.

### 2.5 Diagnostic model for AMI core genes

The diagnostic performance of individual genes in distinguishing between CAD and STEMI samples was evaluated with the “pROC” package (Zhong et al., 2024). Receiver operating characteristic (ROC) curves were plotted, and the area under the curve (AUC) with its 95% confidence interval (95% CI) was calculated for each gene. A multivariable logistic regression model was constructed with the “rms” package (Yan et al., 2024), incorporating the three core genes as predictor variables to discriminate disease status. To achieve quantitative individual risk prediction, a nomogram was plotted via the nomogram function, using a probability scale (0.1–0.99) to intuitively depict the risk contribution conferred by each gene’s expression level. Internal validation of the model was performed with the bootstrap method with 1000 resamples to assess its calibration ability. The model’s discriminative power was also evaluated through ROC analysis, reporting the AUC and its 95% CI to reflect overall predictive accuracy.

Furthermore, the combined model was applied to an external validation set (GSE59867). Sample-wise predicted probabilities were algorithmically derived by reapplying the established model pipeline. A ROC curve was then plotted to evaluate the model’s performance in the external cohort. The diagnostic efficacy and robustness of the model were comprehensively evaluated by comparing the AUC values and 95% CI across different datasets.

### 2.6 scRNA-seq data processing for AMI

Stringent cell filtering was implemented to secure high-quality data for subsequent analyses. Cells were removed if they met any of the following conditions: expressing fewer than 200 or more than 6000 genes, having a UMI count below 500 or above 30,000, or containing mitochondrial gene transcripts exceeding 20% of the total transcripts. Following filtering, the data were normalized and scaled with the NormalizeData and ScaleData functions. The top 2000 highly variable genes were selected for downstream analysis via the FindVariableFeatures function. To reduce dimensionality, principal component analysis was conducted on these genes with the RunPCA function.

To integrate cells from different datasets and mitigate batch effects, the RunHarmony function from the “Harmony” package was used to project the cells into a shared embedding space. Selection of principal components (PCs) for clustering and UMAP was guided by a combination of the two strategies below: ① the cumulative explained variance of selected PCs exceeded 80%, and the variance contributed by any additional PC fell below 5%; ② the optimal number of PCs was defined as the point where the variance drop between consecutive PCs first became less than 0.1%. Based on these criteria, the first 23 PCs were selected for UMAP visualization. Cell clusters were identified using the FindClusters function with a resolution parameter set to 0.2.

Marker genes for each cell cluster were identified with the FindAllMarkers function, employing the Wilcoxon rank-sum test with a threshold of logfc. threshold >0.25 and an adjusted p-value <0.05. Finally, the cell types for each cluster were annotated by referencing canonical peripheral blood cell markers documented in the CellMarker 2.0 database (http://bio-bigdata.hrbmu.edu.cn/CellMarker/).

### 2.7 Enrichment analysis

Gene Ontology (GO) analysis and Kyoto Encyclopedia of Genes and Genomes (KEGG) pathway enrichment analysis were conducted with the “clusterProfiler” package (Wu et al., 2021). The Benjamin-Hochberg method was applied to correct for multiple testing. Enriched GO terms and KEGG pathways for the module or cell-type marker genes were considered significant at an adjusted *p*-value <0.05.

### 2.8 Pseudotime analysis

Pseudotime analysis of the gene expression matrix from myeloid cells was conducted using the “Monocle” package (Wei et al., 2024). Monocle can order cells based on expression changes during differentiation and reconstruct potential branched biological processes. The differentialGeneTest function was used to identify DEGs for each cell subset, which served as a feature gene set to distinguish between different myeloid cell fates. Subsequently, the DDRTree method was employed for dimensionality reduction of the data, and the plot_cell_trajectory function was used to visualize the distribution of cells along the trajectory and the structure of the minimum spanning tree. Finally, using cells from the low-risk group as the starting point, the orderCells function was applied to construct the differentiation trajectory of myeloid cells under different risk states, thereby revealing their differentiation pathways across varying risk conditions.

### 2.9 Cell cultivation

Human cardiomyocyte AC16 cells were purchased from the American Type Culture Collection (ATCC, CRL-3568, United States). The cells were cultured in DMEM medium (Beyotime, C0891-500ml, China) supplemented with 10% FBS. Cultures were maintained in a humidified incubator (37 °C, 5% CO_2_).

### 2.10 Oxygen-glucose deprivation/reperfusion (OGD/R) cell model

AC16 cells were initially cultivated under normal conditions (37 °C, 20% O_2_) in DMEM medium. To simulate ischemia, the cells were rinsed with PBS, and the culture medium was replaced with glucose- and serum-free DMEM. The cells were then incubated for 6 h in a hypoxic environment (37 °C, 2% O_2_). Reperfusion was simulated for 6 h by replacing the medium with complete DMEM (10% FBS) and incubating under normoxic conditions (20% O_2_, 37 °C). Control cells were incubated in a standard incubator (37 °C, 20% O_2_).

### 2.11 Lentiviral transduction and cell treatment

Lentiviral vectors targeting ADAP2 (shADAP2) and the corresponding negative control (shNC) were purchased from GenePharma (Shanghai, China). Following an 8-h incubation with the viral particles, the medium was refreshed with complete medium to mitigate viral cytotoxicity. We initiated puromycin selection at 72 h post-transduction by switching to medium supplemented with 2 μg/mL puromycin (MCE, HY-K1057, United States). Selection continued for about 10 days, with fresh puromycin-containing medium replenished every 2–3 days, until cell death in the control group and the formation of viable, resistant colonies in the experimental group were confirmed. The surviving positive cells were expanded and subsequently passaged with a maintenance medium containing 1 μg/mL puromycin to obtain a stably transduced cell line for subsequent functional studies.

In a rescue experiment, recombinant human IL-17A (rhIL-17A) at a concentration of 100 μg/L (Univ, 2275-IL-050, China) was applied to AC16 cells with stable ADAP2 knockdown. Based on the OGD/R model, these four treatment groups were established: shNC control under OGD/R conditions, shADAP2 knockdown group, rhIL-17A-treated group, and the shADAP2 + rhIL-17A co-treatment group. rhIL-17A was administered at a final concentration of 100 μg/L. Treatment commenced simultaneously with the start of reperfusion and continued for the full 6 h.

### 2.12 qRT-PCR

Total RNA was extracted from cells with TRIzol reagent (Invitrogen, 15596026CN, United States). Reverse transcription of 1 μg RNA into cDNA was performed with a commercial kit (Vazyme, R233-01, China) following the provided protocol. Using the cDNA as a template, amplification reactions were performed on a real-time quantitative PCR system with SYBR Green premix (Vazyme, Q712-02, China). Relative gene expression (target genes vs. β-actin) was quantified with the comparative 2^−ΔΔCt^ method. Primer sequences are shown in Table 1.

**TABLE 1:** Primer Sequences for qRT-PCR.

Gene	Primer	Sequence (5’ → 3’)
ADAP2	Forward	5’-TGC​GAC​TTG​ACT​TCT​GGG​AC-3’
	Reverse	5’-GGC​CTG​GGG​GAT​GTA​GTA​GA-3’
TNFα	Forward	5’- CAA​GGA​CAG​CAG​AGG​ACC​AG-3’
	Reverse	5’- TCC​TTT​CCA​GGG​GAG​AGA​GG-3’
IL-6	Forward	5’- GGA​GCA​GTG​GCT​TCG​TTT​CAT -3’
	Reverse	5’-GAG​GAT​GGC​TGG​ATG​GTT​TCA -3’
IL-1β	Forward	5’-CAG​AAG​TAC​CTG​AGC​TCG​CC -3’
	Reverse	5’- AGA​TTC​GTA​GCT​GGA​TGC​CG -3’
β-actin	Forward	5’-CTG​GAA​CGG​TGA​AGG​TGA​CA-3’
	Reverse	5’-GTC​CTC​GGC​CAC​ATT​GTG​AA-3’

### 2.13 Cell Counting Kit-8 (CCK-8) assay

Cell viability was assessed with the Enhanced CCK-8 (Elabscience, E-CK-A362, China). Cells were seeded in 96-well plates at 1 × 10^4^ cells per well. After the respective treatments, 10 µL of the CCK-8 solution was added to each well. Following a 2-h incubation at 37 °C, the absorbance of each well was measured at 450 nm with a microplate reader.

### 2.14 Cell apoptosis assay

Cell apoptosis was detected with an Annexin V-FITC/PI Apoptosis Detection Kit (Elabscience, E-CK-A211, China). Probably 1 × 10^6^ cells were collected and rinsed twice with PBS. The cells were resuspended in 300 µL of pre-chilled 1× Annexin V binding buffer. 5 μL of Annexin V-FITC dye and 10 µL of PI staining solution were added to the cell suspension. At room temperature and protected from light, the samples were incubated for 10 min following gentle mixing. We immediately acquired the data on a flow cytometer following the reaction.

### 2.15 Terminal deoxynucleotidyl transferase dUTP nick end labeling (TUNEL) staining

Apoptosis in cardiomyocytes was detected with the TUNEL Apoptosis Detection Kit (Elabscience, E-CK-A322, China). Fixed cells were stained with the TUNEL reagent to label apoptotic cells. Then cell nuclei were counterstained with DAPI. The apoptosis rate was quantified as the percentage of TUNEL-positive cells relative to DAPI-positive cells in randomly selected microscopic fields.

### 2.16 Western blot analysis

Total cellular proteins were extracted with RIPA lysis buffer (Beyotime, P0013B, China) supplemented with protease inhibitors on ice. Protein concentration was determined with a BCA Protein Assay Kit (Beyotime, P0009, China). Equal amounts of protein samples were separated by SDS-PAGE and transferred onto PVDF membranes. We blocked the membrane with 5% skimmed milk and then incubated it with the target primary antibody overnight at 4 °C. The primary antibodies used were as follows: anti-IL-17A (Proteintech, 82905-1-RR, Rabbit, 1:2500), anti-IL-17RA (Proteintech, 32055-1-AP, Rabbit, 1:1500), anti-Act1 (Proteintech, 26692-1-AP, Rabbit, 1:1000), and anti-β-actin (Proteintech, 20536-1-AP, Rabbit, 1:5000). Incubation with HRP-labeled goat anti-rabbit IgG secondary antibody (Beyotime, A0208, China; 1:5000) was followed for 1 h at room temperature. Finally, protein bands were visualized with an ECL chemiluminescence kit (Beyotime, P0018S, China) on a Bio-Rad imaging system. Target protein expression was normalized to β-actin for comparative analysis.

### 2.17 Statistical analysis

The data are presented as the mean ± SD, with each group comprising at least three independent biological replicates. GraphPad Prism (version 8.0) was applied in all statistical analyses after verifying the assumptions of normality and homogeneity of variance. Statistical significance was assessed: Student’s *t*-test for two-group comparisons and one-way ANOVA for multi-group comparisons. The p-value <0.05 was statistically significant.

## 3 Results

### 3.1 Identification of core genes in the peripheral blood of AMI patients

**FIGURE 1:**
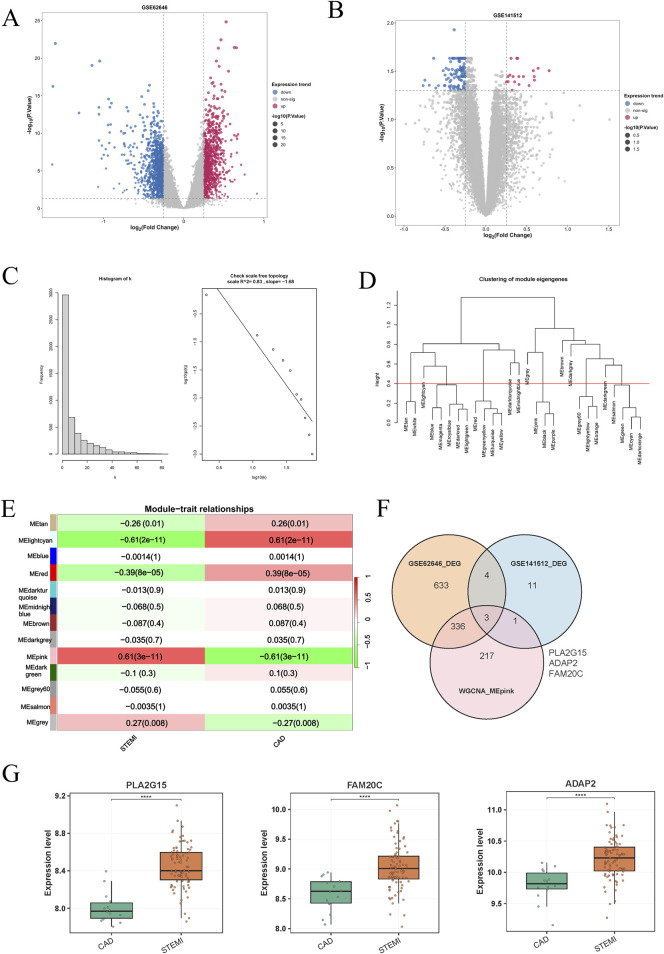
Identification of core genes in AMI peripheral blood. **(A,B)** Volcano plots of AMI-associated differentially expressed genes (DEGs) in the GSE62646 **(A)** and GSE141512 **(B)** datasets. **(C)** Analysis of scale-free topology fit for WGCNA, illustrating the distribution of network connectivity. **(D)** Gene co-expression module clustering dendrogram. Modules were determined via dynamic tree cutting and merging (mergeCutHeight = 0.4). **(E)** Heatmap displaying the correlation of co-expression modules with AMI. The MEpink module showed a significant positive correlation. **(F)** Venn diagram identifying the three core genes through the intersection of the MEpink module genes and DEGs from GSE62646 and GSE141512. **(G)** Comparison of ADAP2, FAM20C, and PLA2G15 expression levels in peripheral blood between STEMI and CAD patients.

Limma analysis of AMI patient peripheral blood yielded 976 DEGs from the GSE62646 dataset and 19 from the GSE141512 dataset (Figures 1A,B). WGCNA was used to assess the connectivity distribution among all genes in the dataset. The results indicated that gene connectivity approximately followed a normal distribution, without genes exhibiting abnormally high connectivity. The scale-free topology fit index exceeded 0.8, and its R^2^ value was close to 1, showing that the network possessed good scale-free properties (Figure 1C). Then, highly correlated modules were integrated by setting the parameter mergeCutHeight = 0.4, ultimately identifying a co-expression module associated with AMI (Figure 1D). The MEpink module exhibited a strong positive association with AMI status (0.61, 3e-11) (Figure 1E). Finally, we intersected the genes from the WGCNA-derived MEpink module with the DEGs from the GSE62646 and GSE141512 datasets, yielding the AMI core genes PLA2G15, ADAP2, and FAM20C (Figure 1F). Furthermore, validation analysis using transcriptome data confirmed that ADAP2, FAM20C, and PLA2G15 were all markedly upregulated in the peripheral blood of AMI patients (Figure 1G).

### 3.2 Evaluation of the diagnostic value of core genes and construction of a diagnostic model

**FIGURE 2:**
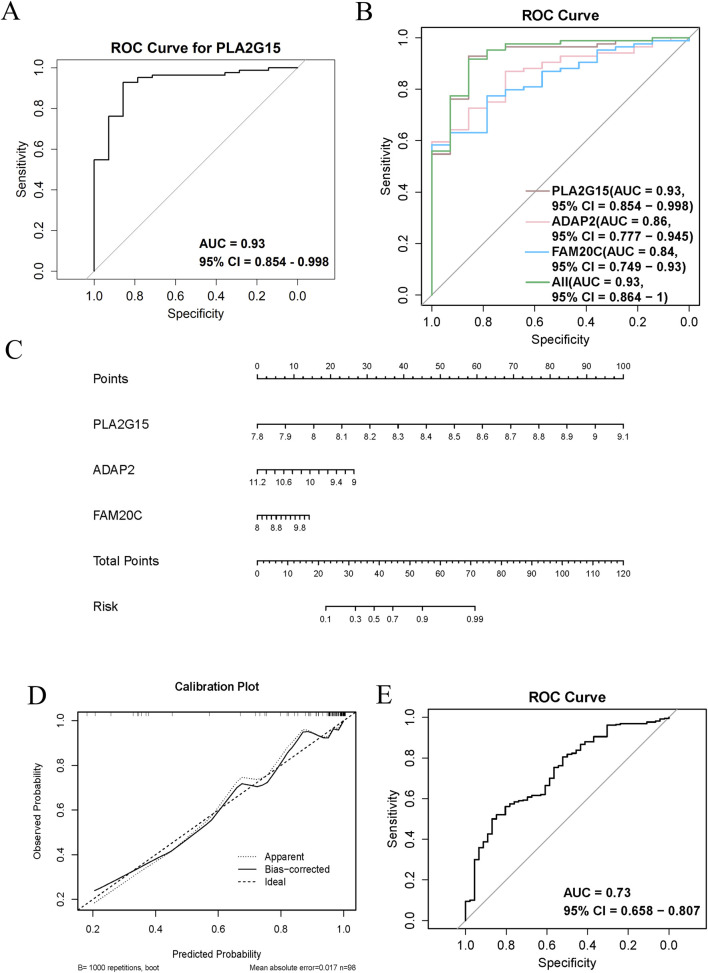
Evaluation of the diagnostic value of core genes and construction of the diagnostic model. **(A)** ROC curves for individual core genes PLA2G15 in the GSE62646 training set. **(B)** ROC curve of the multivariable logistic regression combined diagnostic model based on ADAP2, FAM20C, and PLA2G15 in the GSE62646 training set. **(C)** Nomogram for predicting the risk probability of AMI using the core genes. **(D)** Calibration curve of the nomogram. **(E)** ROC curve of the combined diagnostic model in the GSE59867 validation set.

We began by assessing the diagnostic potential of each core gene. Individual diagnostic models were constructed, and their performance was quantified by plotting and analyzing ROC curves. The ROC curves demonstrated that ADAP2, FAM20C, and PLA2G15 exhibited good discriminatory ability in the GSE62646 training set, with AUC values of 0.86, 0.84, and 0.93, respectively (Figure 2A; Supplementary Figure S1). Integration of the three core genes into a multivariable logistic regression model markedly enhanced diagnostic accuracy, yielding a combined AUC of 0.93 and indicating superior discrimination (Figure 2B). A nomogram was constructed from the model, offering a visual interpretation of gene-specific risk contributions and enabling individualized risk estimation (Figure 2C). Internal validation via bootstrap resampling showed excellent calibration of the nomogram, with predicted risks closely aligning with actual outcomes, underscoring the model’s robustness (Figure 2D). To validate the generalizability of the model, it was further tested on an independent external validation set (GSE59867), where the combined model achieved an AUC of 0.73, indicating maintained good diagnostic accuracy across different cohorts (Figure 2E). Taken together, our integrated three-gene model showed robust diagnostic performance for AMI upon internal and external validation.

### 3.3 scRNA-seq immune profiling of peripheral blood from AMI patients

**FIGURE 3:**
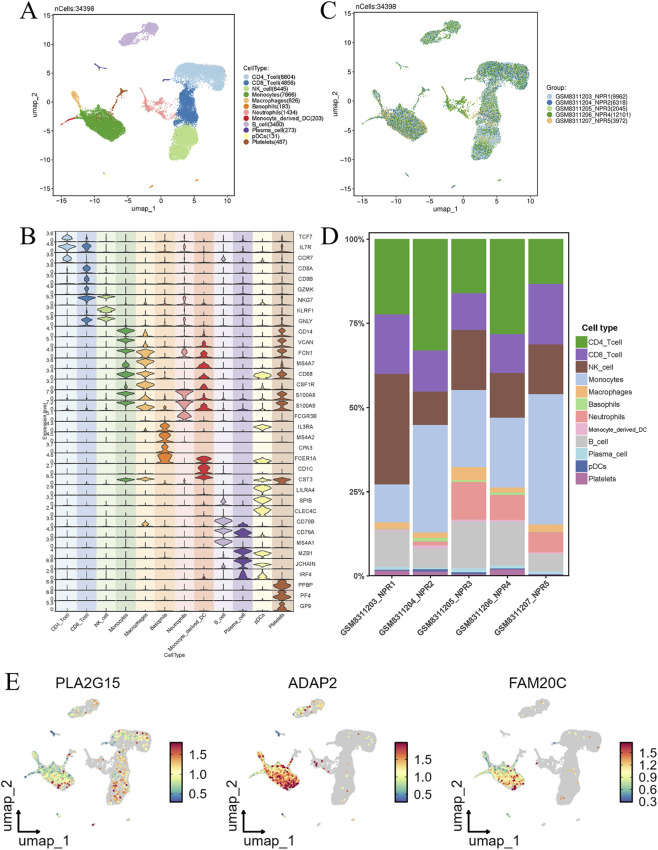
Immune Cell Atlas of AMI Peripheral Blood and Expression Characteristics of Core Genes. **(A)** UMAP plot showing dimensionality reduction and visualization of all immune cells based on scRNA-seq data. **(B)** Expression levels of canonical marker genes across the identified cell subsets. **(C)** Distribution of sample origins within the UMAP space, illustrating the cellular composition from different samples. **(D)** Bar plot showing the proportional distribution of different cell types within each sample. **(E)** UMAP plots displaying the expression distribution of the core genes ADAP2, FAM20C, and PLA2G15 across all immune cells.

The single-cell data were initially processed through unsupervised clustering followed by cell type identification to characterize the immune cell repertoire in AMI peripheral blood. This analysis defined 11 distinct cell subsets with a platelet population. These encompassed major immune cell types, including myeloid cells (monocytes, macrophages, neutrophils, basophils, monocyte-derived dendritic cells, and plasmacytoid dendritic cells) and lymphoid cells (CD4^+^ T cells, CD8^+^ T cells, natural killer cells, B cells, and plasma cells) (Figures 3A,B). Further analysis of the cellular composition across samples revealed that myeloid cells predominated (Figures 3C,D). Notably, the composition of immune cells exhibited evident inter-sample heterogeneity. Specifically, lymphocytes predominated in sample GSM8311203_NPR1, whereas myeloid cells constituted the major cell population in the other four samples (Figure 3D). Such heterogeneity may arise from biological discrepancies linked to the time interval between blood collection and the onset of acute myocardial infarction (AMI), given that acute inflammatory responses characterized by myeloid cell mobilization undergo dynamic changes throughout disease progression. Despite the distinct differences in immune cell profiles across specimens, the expression patterns of hub genes remained consistent in all tested samples (Supplementary Figure S2), which further validates the robustness of findings obtained in this study. A distinct expression pattern was observed upon mapping: ADAP2 and FAM20C expression was predominantly restricted to myeloid lineages, in contrast to the broad distribution of PLA2G15 across multiple immune cell subsets (Figure 3E).

### 3.4 The roles of ADAP2 and FAM20C in myeloid cells

**FIGURE 4:**
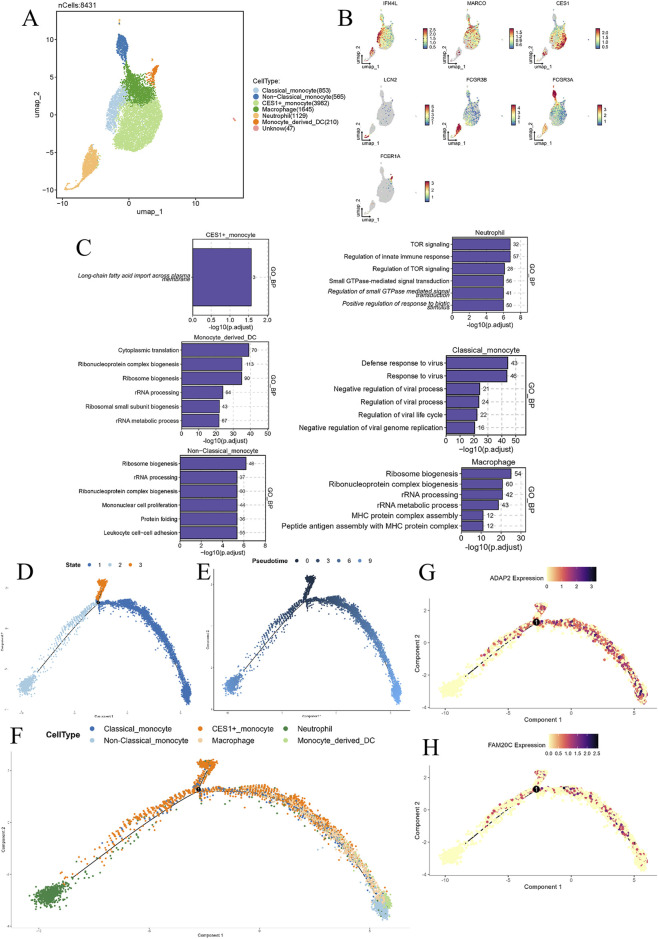
The roles of ADAP2 and FAM20C in myeloid cells. **(A)** UMAP plot showing the sub-clustering of myeloid cell subsets. **(B)** Expression distribution of characteristic marker genes across the myeloid cell subsets. **(C)** Results of GO biological process enrichment analysis for each myeloid cell subset. **(D)** Distribution of myeloid cell states along the differentiation trajectory. **(E)** Trajectory path of cells based on pseudotime. **(F)** Trajectory illustrating cell differentiation towards terminal states, corresponding to specific myeloid cell subtypes. **(G,H)** Expression patterns of the core genes ADAP2 **(G)** and FAM20C **(H)** along the myeloid cell differentiation trajectory.

To dissect the myeloid compartment at higher resolution, we subset and re-clustered these cells, aiming to elucidate the specific function of ADAP2 and FAM20C in differentiation trajectories. Based on the expression of specific marker genes, myeloid cells were further subdivided into multiple subsets with distinct functional characteristics (Figures 4A,B). GO enrichment analysis showed subset-specific functional enrichments, primarily involving biological processes such as ribosome and protein synthesis, antigen presentation, and immune regulation (Figure 4C). Pseudotime analysis was used to reconstruct the differentiation trajectory of myeloid cells, revealing that the cells ultimately converged into a major terminal state (State 1), corresponding to macrophage and monocyte lineages (Figures 4D–F). Intriguingly, ADAP2 and FAM20C expression exhibited a progressive increase along the differentiation trajectory toward terminal effector phenotypes, implicating these genes in the regulation of myeloid maturation (Figures 4G,H).

### 3.5 ADAP2 exacerbates OGD/R-induced cardiomyocyte injury

**FIGURE 5:**
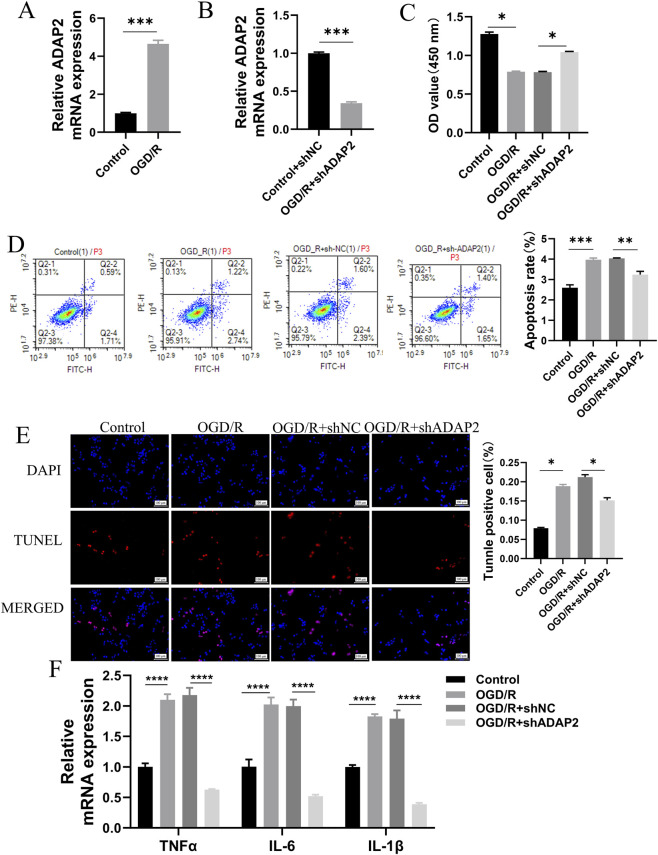
ADAP2 exacerbates OGD/R-induced cardiomyocyte injury. **(A)** mRNA expression level of ADAP2 in AC16 cells after OGD/R treatment. **(B)** Successful construction of the ADAP2-knockdown cell model under OGD/R conditions. **(C)** Effect of ADAP2 knockdown on cell proliferation under OGD/R conditions, as detected by the CCK-8 assay. **(D,E)** Effects of ADAP2 knockdown on cell apoptosis under OGD/R conditions, assessed by Annexin V-FITC/PI flow cytometry **(D)** and TUNEL staining **(E)**. **(F)** Effect of ADAP2 knockdown on the mRNA expression of inflammatory factors TNF-α, IL-6, and IL-1β under OGD/R conditions, as detected by qRT-PCR.

Although our study primarily focused on peripheral blood samples, ADAP2 is upregulated in AMI and has been reported to promote cardiomyocyte hypertrophy, contributing to pathological remodeling by enhancing β1-integrin clustering and microtubule detyrosination (Berthiaume et al., 2025; Osmak et al., 2020). However, the research on ADAP2 in AMI remains insufficiently explored. We investigated its function in human AC16 cardiomyocytes. To examine the function of ADAP2 in AMI, we first established an OGD/R model in AC16 cells. A significant increase in ADAP2 mRNA levels was observed in cells subjected to OGD/R compared to those under normal conditions (Figure 5A). Based on this, we successfully constructed an OGD/R cell model with ADAP2 knockdown (Figure 5B). Phenotypic analysis revealed that OGD/R treatment markedly inhibited cell proliferation, promoted apoptosis, and markedly upregulated the mRNA expression of the inflammatory factors TNF-α, IL-6, and IL-1β. Knockdown of ADAP2 substantially reversed the OGD/R-induced cellular damage and inflammatory response (Figures 5C–F). Taken together, ADAP2 is activated during myocardial ischemia-reperfusion injury and is a key pathogenic molecule in the progression of AMI.

### 3.6 ADAP2 mediates OGD/R-induced cardiomyocyte injury by regulating the IL-17 signaling pathway

**FIGURE 6:**
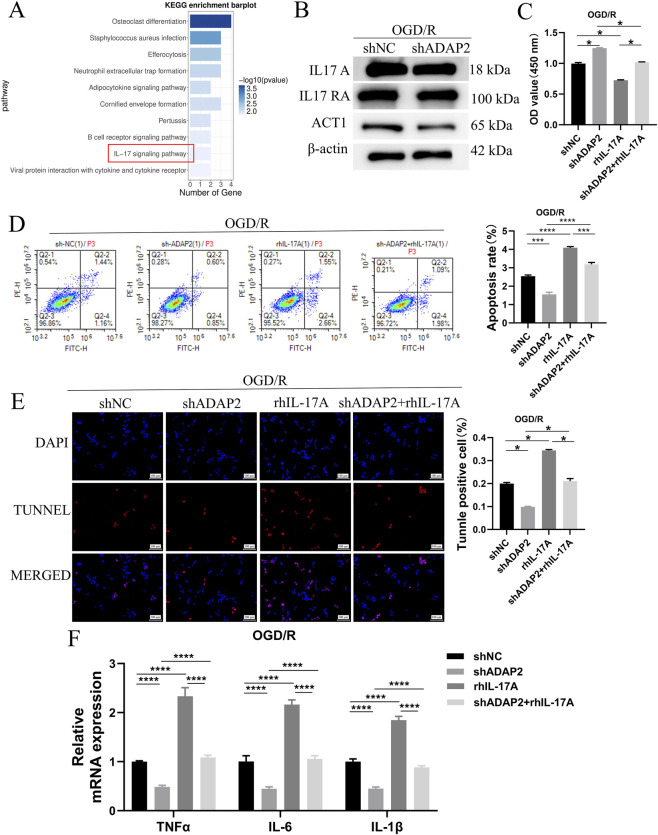
ADAP2 mediates OGD/R-induced cardiomyocyte injury by regulating the IL-17 signaling pathway. **(A)** KEGG pathway enrichment analysis of ADAP2. **(B)** Western blot analysis of the protein expression levels of key IL-17 signaling pathway molecules (IL-17A, IL-17RA, and ACT1) after ADAP2 knockdown. **(C)** Changes in cell proliferation viability under OGD/R conditions across different treatment groups, as measured by the CCK-8 assay. **(D,E)** Changes in the apoptosis rate under OGD/R conditions across different treatment groups, detected by flow cytometry **(D)** and TUNEL staining **(E)**. **(F)** mRNA expression levels of the inflammatory factors TNF-α, IL-6, and IL-1β in different treatment groups under OGD/R conditions, as detected by qRT-PCR.

To delineate the pathogenic mechanisms of ADAP2 in AMI, functional enrichment analysis was undertaken, pinpointing a strong association with the IL-17 signaling pathway (Figure 6A). Research indicated that the IL-17 signaling pathway is instrumental in myocardial infarction (Jain, 2022). Following myocardial infarction, the expression of IL-17 is persistently increased (Ren et al., 2020), suggesting its potential involvement in the pathogenesis of AMI. Consequently, we focused our investigation on this pathway. We first examined the effect of ADAP2 knockdown on the expression of key molecules in the IL-17 signaling pathway, namely, IL-17A, IL-17RA, and ACT1. Their protein levels were markedly decreased upon ADAP2 knockdown (Figure 6B). To further elucidate the functional link between ADAP2 and this pathway, we established four treatment groups in OGD/R-treated AC16 cells: siNC control, siADAP2, rhIL-17A treatment, and siADAP2 combined with rhIL-17A co-treatment, and then assessed their functional changes. The results showed that under OGD/R conditions, knockdown of ADAP2 markedly promoted cell proliferation, whereas exogenous addition of rhIL-17A reversed this effect (Figure 6C). Similarly, the suppression of cell apoptosis and the downregulation of inflammatory factors (TNF-α, IL-6, IL-1β) induced by ADAP2 knockdown were restored to baseline levels upon co-treatment with rhIL-17A (Figures 6D,E). Thus, ADAP2 functions to exacerbate cardiomyocyte damage under OGD/R conditions through modulation of IL-17 signaling.

## 4 Discussion

By integrating multi-omics and systems biology, this research reveals pivotal molecular characteristics and the immune microenvironment in AMI peripheral blood. We identified three core genes, PLA2G15, ADAP2, and FAM20C, and constructed a multi-gene combined model with favorable diagnostic performance. The role and molecular mechanism of ADAP2 in cardiomyocyte damage were further revealed through *in vitro* studies, offering fresh insights into the potential mechanisms of AMI development.

ADAP2 encodes the ArfGAP with dual PH domains 2 protein, which is significant in cardiac development. Given its established role in cardiac development, dysfunction of this gene product is implicated in the pathogenesis of cardiovascular anomalies associated with NF1 microdeletion syndrome (Venturin et al., 2014). Furthermore, ADAP2 is a key hub gene contributing to the initiation and progression of atherosclerosis (Wu et al., 2024). Despite the absence of studies directly connecting FAM20C with AMI, it can prevent fatal ventricular arrhythmias by phosphorylating the Ser96 site of the HRC protein, thereby regulating cardiomyocyte sarcoplasmic reticulum calcium cycling (Pollak et al., 2017). Importantly, animal model studies have demonstrated that cardiomyocyte-specific knockout of FAM20C leads to progressive heart failure in mice following aging or pressure overload. FAM20C-mediated phosphorylation of calsequestrin 2 and STIM1 is essential for maintaining sarcoplasmic reticulum Ca^2+^ homeostasis, and dysregulated Ca^2+^ signaling represents a central component of cardiac pathophysiology (Pollak et al., 2018). PLA2G15 is a calcium-independent phospholipase that functions in lysosomes, primarily responsible for degrading phospholipids and sphingolipids, and is important in lipid metabolism and immune-inflammatory regulation (Nyame et al., 2025). Studies on PLA2G15-deficient mice reveal that this enzyme exerts pivotal functions in the catabolism of phospholipids within pulmonary surfactant and the modulation of macrophage-driven inflammatory responses. Mice with PLA2G15 deficiency develop exacerbated tissue fibrosis and impaired lipid clearance after injury, which underscores the comprehensive roles of this enzyme in sustaining immune and inflammatory homeostasis (Shayman and Tesmer, 2019; Kwak et al., 2025). Despite its implicated role, the precise function of PLA2G15 in cardiovascular pathology is not well defined. Our discovery paves the way for future functional studies. In the diagnostic model, these three genes demonstrated high diagnostic efficacy when analyzed individually. Their combined model also maintained robust performance in both the training set and external validation set. Thus, this gene panel holds promise as a minimally invasive blood biomarker. The construction of the nomogram significantly enhances the clinical utility of the model, thereby expanding its potential utility in translational research.

Research has confirmed that ADAP2 expression is markedly upregulated in patients with myocardial infarction and drives pathological hypertrophy in adult cardiomyocytes by promoting β1-integrin clustering and microtubule detyrosination (Berthiaume et al., 2025; Osmak et al., 2020). Given the reported involvement of ADAP2 in integrin-mediated hypertrophic responses in cardiomyocytes, we further investigated its function in the OGD/R model using AC16 cells. ADAP2 was confirmed to be upregulated upon OGD/R, and its knockdown ameliorated the associated cellular injury, including suppressed proliferation, enhanced apoptosis, and elevated inflammatory response. Thus, ADAP2 exacerbates cellular damage following ischemia-reperfusion.

IL-17 is a pleiotropic cytokine produced by Th17 cells and others, serving as a pivotal link between innate and adaptive immunity and a major in cardiovascular diseases (Ma et al., 2025). Inflammation is a key driver of adverse cardiac remodeling following AMI (Yong et al., 2025). Specifically, IL-17 exacerbates inflammatory responses and myocardial injury by mediating the migration and activation of monocytes, a process involving Toll-like receptor 4 and the production of IL-6 (Sun et al., 2021). Furthermore, in a mouse model of AMI induced by permanent ligation of the left anterior descending coronary artery, genetic deficiency of IL-17A significantly reduced myocardial fibrosis, apoptosis, and infarct size while improving cardiac function. Conversely, exogenous supplementation of IL-17A aggravated ventricular remodeling and worsened cardiac function (Zhou et al., 2014). Mechanistically, KEGG enrichment analysis suggested a close association between ADAP2 and the IL-17 signaling pathway. We confirmed that knockdown of ADAP2 suppressed the expression of IL-17A, IL-17RA, and ACT1, and that treatment with exogenous IL-17A reversed the protective effects conferred by ADAP2 knockdown. This indicates that ADAP2 likely exacerbates cardiomyocyte injury by regulating the IL-17 signaling pathway.

Our single-cell transcriptomic analysis revealed that myeloid cells predominate within the immune cell composition in the peripheral blood of AMI patients. Myeloid cells comprise macrophages, dendritic cells, monocytes, and granulocytes (van Vlerken-Ysla et al., 2023). AMI is an inflammatory response mediated by monocytes and T cells. Prior research indicates that monocytes are actively recruited from the circulation into atherosclerotic plaques, where they differentiate into macrophages and ultimately form foam cells (Qian et al., 2022). Macrophages are central players in AMI. They clear necrotic cells and regulate the inflammatory response and engage in “crosstalk” with other cardiac cells, such as cardiomyocytes, fibroblasts, and vascular endothelial cells, and secrete mediators like exosomes, collectively coordinating key processes including cardiac repair, fibrosis, angiogenesis, and lymphangiogenesis (Jian et al., 2023). Furthermore, different macrophage subsets exert distinct functions in AMI. Pro-inflammatory CCR2^+^ macrophages exacerbate inflammation and injury. In contrast, administering small extracellular vesicles derived from M2 macrophages can polarize them toward a pro-reparative M2-like phenotype. These vesicles carry miR-181b-5p, which inhibits inflammation and promotes cardiac repair (Li et al., 2023). In AMI, myeloperoxidase activates monocytes and upregulates surface CCR2 expression, thereby driving the migration and infiltration of splenic monocytes into the ischemic heart region, worsening inflammation and contributing to adverse cardiac remodeling (Peters et al., 2024). In AMI, neutrophils markedly aggravate cardiac damage and thromboinflammation through mechanisms involving their internal NLRP3 inflammasome, which drives IL-1β production, von Willebrand factor release, and NET formation (Heger et al., 2024). We found that the expression of two key core genes, ADAP2 and FAM20C, is highly concentrated within myeloid cell subsets. Thus, they might be key mediators linking myeloid cells to the AMI-associated inflammatory cascade.

This study systematically identified core genes (PLA2G15, ADAP2, and FAM20C) in the peripheral blood of AMI patients by integrating multi-omics data. By building a robust multi-gene diagnostic signature and applying single-cell transcriptomics, we uncovered the roles of these genes in immune regulation, with a focus on myeloid differentiation. However, this study has several limitations. First, all analyses were based on retrospective data from public databases, with limited sample sizes and potential batch effects. Although external validation was performed, further validation using prospective clinical cohorts is lacking. Second, the scRNA-seq data were derived from only five STEMI patients. This small sample size limits the robustness of statistical inferences for cell subsets and the generalizability of biological conclusions. Furthermore, the single-cell data analysis only revealed the expression patterns of the core genes in immune cells; it did not experimentally validate their specific regulatory mechanisms within cell subsets. Finally, the mechanistic link between ADAP2 and the IL-17 pathway was established only *in vitro*. Future work employing animal models is warranted to elucidate its *in vivo* mechanisms and potential crosstalk within the broader signaling network during AMI. Future research should validate the reliability of the diagnostic model using larger-scale clinical cohorts and employ animal models to deeply investigate the specific mechanisms of ADAP2 in AMI, aiming to advance its translation into clinical diagnostics and therapeutic targets. Nevertheless, this study systematically identified core genes with diagnostic potential in the peripheral blood of AMI and constructed a diagnostic model based on them. It revealed a new mechanism by which ADAP2 exacerbates myocardial injury through the IL-17 signaling pathway, thereby furnishing novel insights and a robust database for the mechanistic understanding of AMI.

## Data Availability

The original contributions presented in the study are publicly available. This data can be found here: [https://doi.org/10.5281/zenodo.20793396].
