# Supplementary figures and images for: Application of acute myocardial infarction-related key genes in noninvasive diagnosis: a comprehensive analysis based on transcriptome and single-cell transcriptome
Source: Front Genet. 2026 Jul 2;17:1828693. doi: 10.3389/fgene.2026.1828693 (PMC13372506; doi:10.3389/fgene.2026.1828693)

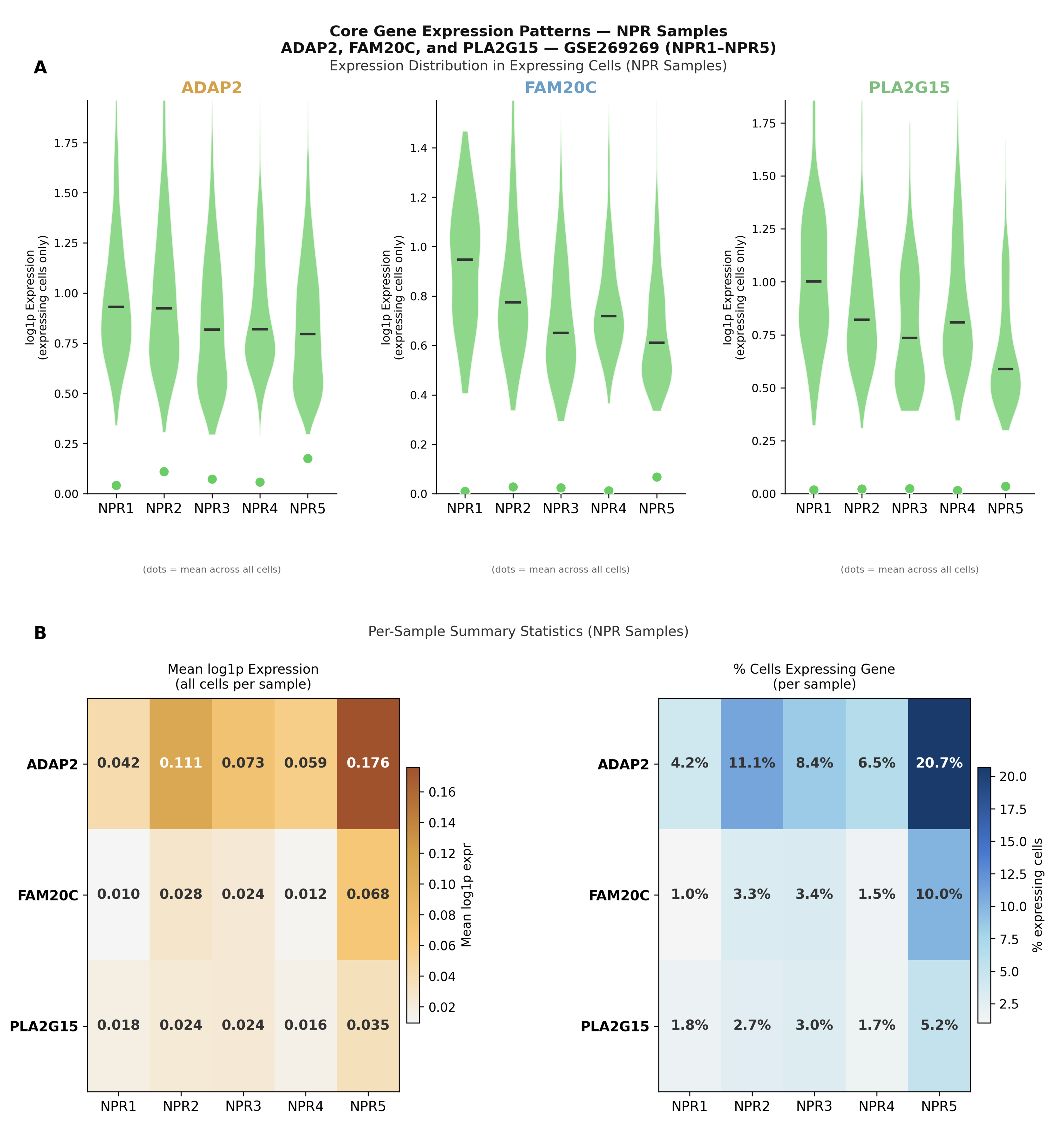

Supplement: Supplementary file 1 [file Image2.tif]

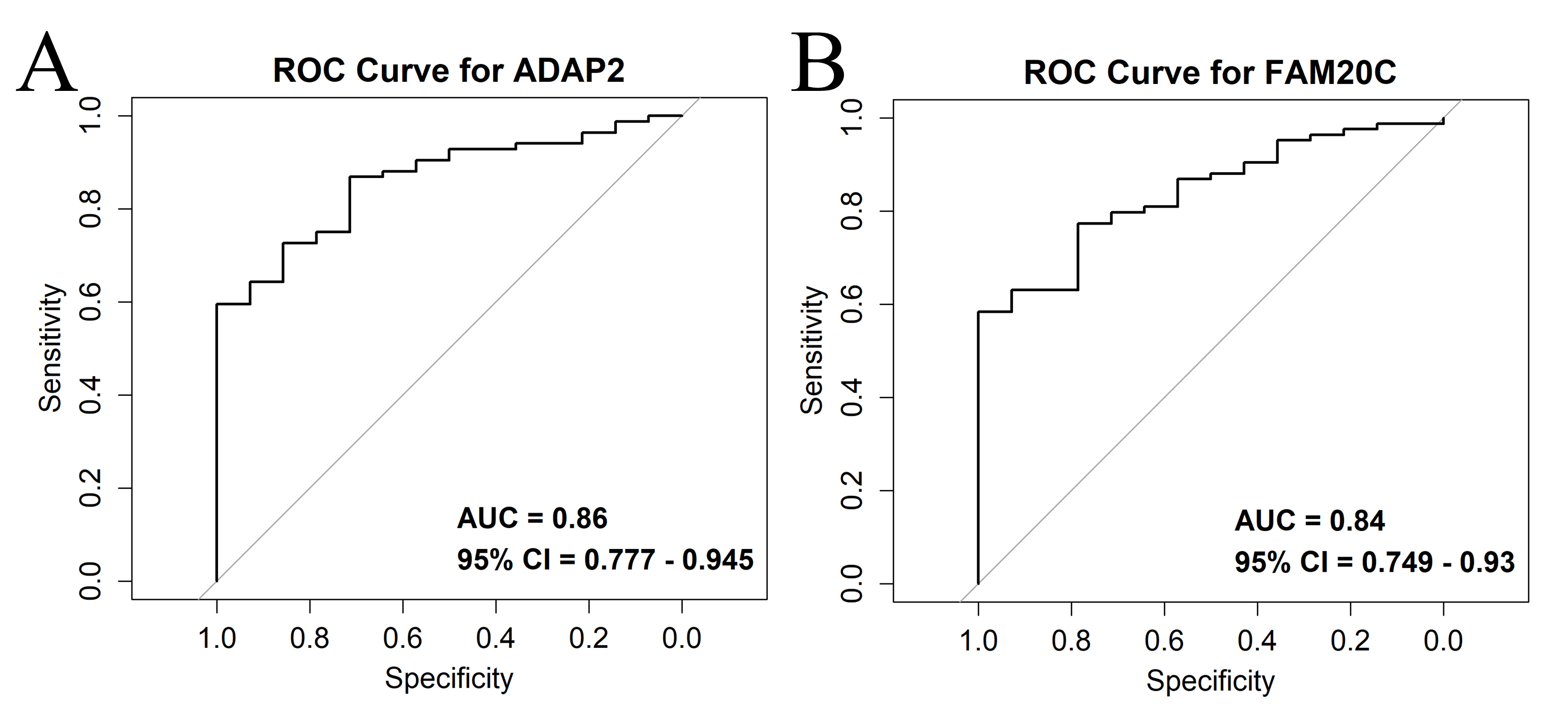

Supplement: Supplementary file 2 [file Image1.tif]
